# Domestication of cattle: Two or three events?

**DOI:** 10.1111/eva.12674

**Published:** 2018-07-23

**Authors:** Daniel Pitt, Natalia Sevane, Ezequiel L. Nicolazzi, David E. MacHugh, Stephen D. E. Park, Licia Colli, Rodrigo Martinez, Michael W. Bruford, Pablo Orozco‐terWengel

**Affiliations:** ^1^ School of Biosciences Cardiff University Cardiff UK; ^2^ Parco Tecnologico Padano (PTP) Lodi Italy; ^3^ Animal Genomics Laboratory UCD School of Agriculture and Food Science, UCD College of Health and Agricultural Sciences University College Dublin Dublin Ireland; ^4^ UCD Conway Institute of Biomolecular and Biomedical Research University College Dublin Dublin Ireland; ^5^ IdentiGEN Ltd. Dublin Ireland; ^6^ Istituto di Zootecnica e BioDNA Centro di Ricerca sulla Biodiversità e sul DNA Antico Università Cattolica del S. Cuore di Piacenza Piacenza Italy; ^7^ Corporación Colombiana De Investigación Agropecuaria (Corpoica) Centro de investigaciones Tibaitatá Bogotá Colombia

**Keywords:** approximate Bayesian computation, *Bos indicus*, *Bos Taurus*, demographic modeling, domestication history, SNP array

## Abstract

Cattle have been invaluable for the transition of human society from nomadic hunter‐gatherers to sedentary farming communities throughout much of Europe, Asia and Africa since the earliest domestication of cattle more than 10,000 years ago. Although current understanding of relationships among ancestral populations remains limited, domestication of cattle is thought to have occurred on two or three occasions, giving rise to the taurine (*Bos taurus*) and indicine (*Bos indicus*) species that share the aurochs (*Bos primigenius*) as common ancestor ~250,000 years ago. Indicine and taurine cattle were domesticated in the Indus Valley and Fertile Crescent, respectively; however, an additional domestication event for taurine in the Western Desert of Egypt has also been proposed. We analysed medium density Illumina Bovine SNP array (~54,000 loci) data across 3,196 individuals, representing 180 taurine and indicine populations to investigate population structure within and between populations, and domestication and demographic dynamics using approximate Bayesian computation (ABC). Comparative analyses between scenarios modelling two and three domestication events consistently favour a model with only two episodes and suggest that the additional genetic variation component usually detected in African taurine cattle may be explained by hybridization with local aurochs in Africa after the domestication of taurine cattle in the Fertile Crescent. African indicine cattle exhibit high levels of shared genetic variation with Asian indicine cattle due to their recent divergence and with African taurine cattle through relatively recent gene flow. Scenarios with unidirectional or bidirectional migratory events between European taurine and Asian indicine cattle are also plausible, although further studies are needed to disentangle the complex human‐mediated dispersion patterns of domestic cattle. This study therefore helps to clarify the effect of past demographic history on the genetic variation of modern cattle, providing a basis for further analyses exploring alternative migratory routes for early domestic populations.

## 1 INTRODUCTION

Between the late Pleistocene and early Holocene, the most commonly occurring cattle species was the aurochs (*Bos primigenius*), ranging from northern Africa to both the Atlantic and Pacific coasts of Eurasia (Zeuner, 1963). This formerly widespread wild species recently became extinct, with the last recorded herd found in 1627 AD in Poland (Götherström et al., 2005). Similar to sheep and goats, archaeological and genomic evidence suggests that the ancestors of taurine cattle (*Bos taurus*) were domesticated from *Bos primigenius primigenius* in the Fertile Crescent during the Neolithic, more than 10,000 years ago (YA; Bruford, Bradley, & Luikart, 2003; Ajmone‐Marsan, Garcia, & Lenstra, 2010; MacHugh, Larson, & Orlando, 2017). However, approximately 1,500 years later a second domestication event took place in the Indus Valley from *Bos primigenius nomadicus*, separated from the taurine branch ~250–330,000 YA, eventually giving rise to the extant indicine cattle (*Bos indicus*), often also termed zebu cattle (Loftus, MacHugh, Bradley, Sharp, & Cunningham, 1994).

Despite support for the extant distribution of cattle arising from two main domesticated lineages, a third domestication event has been hypothesized to have occurred in north‐east Africa about 8,000–9,000 YA, giving rise to the divergent African taurine cattle. Support for this hypothesis derives from archaeozoological evidence and genetic data from contemporary cattle and aurochs. The archaeozoological evidence is supported by comparative osteological analyses between ancient wild and domestic cattle from Europe, Asia and Africa (Applegate, Gautier, & Duncan, 2001; Grigson, 1991, 2000; Stock & Gifford‐Gonzalez, 2013). Bradley, MacHugh, Cunningham, and Loftus (1996), using maternal mitochondrial DNA (mtDNA), showed that African cattle feature a higher frequency of the T1 mitochondrial haplogroup than is common in other regions, estimated that the separation between African and European taurine ancestors occurred 22,000–26,000 YA (earlier than the Fertile Crescent domestication), and found patterns of population expansions consistent with domestication that were more recent than the corresponding signature of African/European divergence. These results are supported by analyses of extant taurine cattle from Europe, the Middle East and Africa, as well as extinct British aurochs (Troy et al., 2001). Furthermore, evidence from nuclear DNA analyses (Bovine Hapmap Consortium et al., 2009; Hanotte et al., 2002; Pérez‐Pardal et al., 2010) is consistent with a local domestication of African taurine cattle and the subsequent admixture of Near East and the Indus Valley cattle in Africa. However, Bonfiglio et al. (2012) support the Near Eastern origin of the T1 mitochondrial DNA haplogroups and defended that the North African subhaplogroup T1d could have originated in already domesticated cattle shortly after their arrival from the Near East. Along this line, analyses on whole‐genome SNP arrays supported two domestication events for taurine and indicine lineages followed by introgression from wild aurochs in Africa, East Asia and Europe (Decker et al., 2014). Taken together, controversial archaeological and genetic data seem to support both two and three domestication events in cattle; therefore, an analysis using genealogical modelling of alternative scenarios that could give rise to the extant patterns of cattle genetic diversity needs to be addressed.

**Figure 1:**
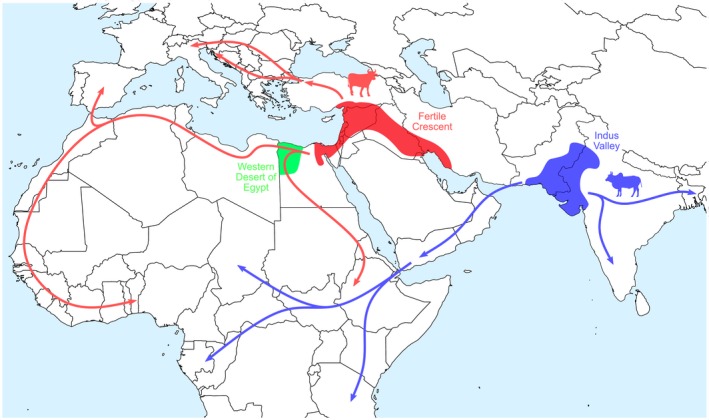
Hypothesized main domestication sites and migration routes of taurine (*Bos taurus*) and indicine (*Bos indicus*) cattle, including the postulated third domestication site in Egypt. Lesser migration routes, such as the dispersal across Europe, are not depicted

Taurine cattle dispersed quickly after their domestication northwest from the Fertile Crescent through Turkey into the Balkans and into northern Italy, either following a Mediterranean coastline or a route partially along the Danube River, and subsequently dispersing across Europe (Figure 1) (Beja‐Pereira et al., 2006; Pellecchia et al., 2007). Taurine cattle may have also migrated along the northern coast of Africa, eventually crossing into the Iberian Peninsula and admixing with local cattle (Figure 1) (Beja‐Pereira et al., 2006; Decker et al., 2009). Similarly, indicine cattle also dispersed far beyond their domestication centre in the Indus Valley, reaching China and much of South‐East Asia (Figure 1) (Ajmone‐Marsan et al., 2010). Additionally, a modern indicine migration via pastoralists into eastern Africa (~2,500–3,500 YA) and subsequently throughout central and southern areas of the continent has been described (Figure 1) (Decker et al., 2014; Hanotte et al., 2002; Payne & Hodges, 1997).

Previous studies using traditional molecular markers (e.g., mitochondrial DNA and microsatellites) generated insights into the genetics underlying the domestication history and postdomestication processes, such as the taurine dispersal patterns in Europe (Beja‐Pereira et al., 2006; Bradley et al., 1996; MacHugh, Shriver, Loftus, Cunningham, & Bradley, 1997). However, the development of newer genomic technologies, such as SNP arrays, has enabled the rapid collection of information for thousands of markers with high precision, facilitating comprehensive surveys of genomewide variation in domestic cattle (Bovine Hapmap et al. 2009).

Here, we collated a data set of ~54,000 SNPs genotyped in 180 cattle populations from across the world, representing both indicine and taurine breeds (Supporting Information Table S1), to characterize the genetic diversity, population structure and demographic history of extant cattle. To disentangle the long‐standing debate around the existence of a third domestication event in north‐east Africa, we used approximate Bayesian computation to model a variety of possible scenarios that could give rise to the extant patterns of cattle genetic diversity, including analyses to determine whether modern cattle derive from two or three domestication events and migratory patterns among breeds.

## 2 MATERIALS AND METHODS

### 2.1 SNP array data

Illumina's BovineSNP50 v.1, v.2 and Bovine High Density BeadChip 770k SNP array data were merged from several previously published studies (Supporting Information Table S1; Bovine Hapmap et al. 2009; Decker et al., 2009; Gautier, Laloë, & Moazami‐Goudarzi, 2010; McTavish, Decker, Schnabel, Taylor, & Hillis, 2013; Decker et al., 2014; Mbole‐Kariuki et al., 2014; Orozco‐terWengel et al., 2015; Park et al., 2015; Iso‐Touru et al., 2016; Upadhyay et al., 2017; Pitt et al., 2018). After removing duplicated copies of individuals, the final data set comprised ~54,000 SNPs in most samples and corresponded to 3,196 individuals representing 180 breeds/populations of both taurine (*n *=* *2,041) and indicine (*n *=* *408) cattle, including hybrids of the two subspecies (Supporting Information Table S1). Additionally, one individual was genotyped from the archaeological remains of a *B. p. primigenius* animal from England (Supporting Information Table S1; Park et al., 2015) and used as an outgroup. The UMD3.1 bovine assembly was used as a reference to map the genomic positions for each SNP. All data sets were merged and quality controlled using PLINK 1.90 (Chang et al., 2015; Purcell et al., 2007). Across all breeds, only autosomal SNPs were retained and any locus with a minor allele frequency less than 5% and a call rate less than 90% was removed, leaving 8,081 SNPs (~8 k) for further analyses.

### 2.2 Genetic variation and population divergence

The molecular inbreeding coefficient (*F*_IS_), and observed (*H*_o_) and expected (*H*_e_ *)* heterozygosities per breed were calculated using PLINK on the breeds with a sample size of at least eight individuals (breeds with lower sample sizes were not included to avoid potentially biased estimates deriving from unrepresentative sample sizes). Welch's *t* test was used to test or differences between *H*_e_ and *H*_o_ within each breed. Admixture v1.3 (Alexander, Novembre, & Lange, 2009) was used to assess population structure in the data for values of K (the number of clusters in the data) between 1 and 150. The predictive accuracy for each K was estimated using the fivefold cross‐validation (CV) procedure implemented in Admixture. For this analysis, we further pruned the 8K data set for linkage disequilibrium (LD) in PLINK, using a sliding window of 50 kb, a step size of 10 SNPs, and randomly removing one SNP from each pairwise comparison with an *r* ^2^ of 5% or higher. Sixty‐one values of K ranging between 1 and 150 were tested. Due to the computational power required for assessing higher values of K, we restricted the analysis to all values of K between 1 and 50, and used intervals of 5 K from 55 to 100, and examined K = 150. The partition solutions were visualized using the POPHELPER R package (Francis, 2017).

Multidimensional scaling (MDS) was calculated in PLINK using Hamming distances to determine the distances between individuals and breeds across 20 dimensions. The first two major axes explaining the highest proportion of the variance in the data set were visualized using R (R Core Team, 2014). *F*_ST_ was also estimated between all pairs of populations featuring more than one sample, and the resultant matrix was used to construct a neighbour‐net tree in SplitsTree v.4.14.4 (Huson & Bryant, 2006).

### 2.3 Approximate Bayesian computation (ABC)

The domestication history of cattle was analysed with approximate Bayesian computation (ABC) as implemented in the software ABCtoolbox (Wegmann, Leuenberger, Neuenschwander, & Excoffier, 2010) with simulations generated using Fastsimcoal2 (Excoffier, Dupanloup, Huerta‐Sánchez, Sousa, & Foll, 2013; Excoffier & Foll, 2011). To reduce the computational power required to perform the analyses, as well as to reduce the effect of ascertainment bias (e.g., Barbato et al., 2017; Kijas et al., 2012; Orozco‐terWengel et al., 2015), the 8K data set was pruned in PLINK as above but with an *r* ^2^ of 2.5%, resulting in 2,202 SNPs (2K). Four breeds were selected for these analyses, a European taurine (TaurEU; Normande [NOR]), an African taurine (TaurAF; Somba [SOM]), an African indicine (ZebuAF; Madagascan Zebu [ZMA]) and an Asian indicine (ZebuAS; Kankraj [KAN]) (Table 1). These breeds were chosen avoiding outlier breeds based on the MDS and neighbour‐net analyses. Two additional breed sets were selected with the same criteria and used as replicates for the ABC analysis to assess that the results obtained were not dependent on the breed set used but rather a general outcome reflective of the domestication history of cattle (Table 1). To ensure the 2K data set retained information about the genealogical history of these breeds, we compared the results of running the Admixture analysis on each set of four breeds using the 2K and 8K data sets. The 2K data set generated qualitatively the same results as the 8K data set. The observed data for each breed set were analysed with Arlequin 3.5 (Excoffier & Lischer, 2010) to produce 34 summary statistics describing the genetic variation within and among breeds (Supporting Information Table S3).

**Table 1:** Breeds sets used on the modelling of domestication history of cattle with approximate Bayesian computation (ABC)

Breed set^a^	TaurEU	TaurAF	ZebuAF	ZebuAS
1	Normande (NOR)	Somba (SOM)	Zebu from Madagascar (ZMA)	Kankraj (KAN)
2	Abondance (ABO)	N'dama (NDA)	Zebu Fulani (ZFU)	Bhagnari (BAG)
3	Montbeliard (MON)	Baoule (BAO)	Zebu Bororo (ZBO)	Dajal (DAJ)

a A breed in each group was selected to represent European taurine (TaurEU), African taurine (TaurAF), African indicine (ZebuAF) and Asian indicine (ZebuAS) groups, avoiding outlier populations based on the multidimensional scaling (MDS) and neighbour‐net analyses.

**Figure 2:**
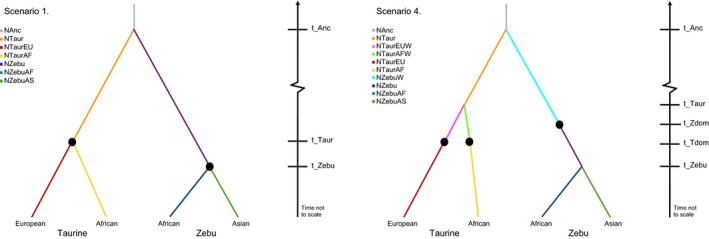
Example of two initial modelled scenarios for determining the domestication history of cattle using approximate Bayesian computation (ABC). Scenario 1 models only two domestication events (black circles) that coincide with the divergence of taurine and indicine cattle. Scenario 4 models three domestication events: one in indicine cattle prior to divergence within the indicine group and two within taurine cattle, after divergence within the taurine group

The underlying model used in the ABC analyses for the four geographically and genetically divergent groups assumed that: (a) ZebuAF and ZebuAS have a common indicine ancestor; (b) TaurEU and TaurAF have a common taurine ancestor; (c) indicine and taurine have a common aurochs ancestor; and (d) bifurcation of aurochs occurred approximately 250,000 YA (Bovine Hapmap et al. 2009; Bradley et al., 1996). Initially, four alternative scenarios were tested to discriminate among the main demographic histories involving two or three domestication events, modelled as bottlenecks, and migratory patterns among breeds (examples shown in Figure 2; full detail in Supporting Information Figure S1). The software ABCtoolbox was used to parametrize the models to be simulated and to control Fastsimcoal2 for the random generation of the 1 million reverse coalescent simulations drawing parameter values from defined prior distribution ranges for each scenario (Supporting Information Table S2). Based on a set of 5,000 randomly selected simulations, Spearman's rank correlations between the 34 summary statistics were calculated to test for the presence of correlations between summary statistics. After Bonferroni correction, summary statistics with consistent significant negative or positive correlations to others were removed from further analyses, reducing the set of statistics for the ABC analysis from 34 to 17. The fitting of the simulations to the observed data was further determined by identifying if each of the summary statistics for the observed data fell within the 95% quantiles of the distribution of the corresponding summary statistics calculated on the simulated data for each scenario. Posterior estimates for each parameter in each scenario were obtained with ABCtoolbox using the 1,000 simulations that produced summary statistics closest to the observed data. The retained simulations were compared to the observed data under a generalized linear model (GLM) to produce a posterior probability *p*‐value. The *p*‐value is the proportion of simulations that have a smaller or equal likelihood to the observed data; larger *p*‐values represent a better fitting GLM and therefore a good match between the simulated and observed scenarios. Different scenarios were compared with Bayes factors (BF), measured as the quotient of the marginal density (MD) of one scenario divided by the MD of an alternative one; a BF greater than 3 is sufficient to reject the alternative scenario (Wegmann et al., 2010). Upon selecting the best‐fitting model of the initial four scenarios proposed, alternative scenarios were hypothesized using this as a base model, and the process was repeated multiple times until all 15 scenarios were tested (Supporting Information Figure S1). More complex scenarios were not tested to avoid uninformative overparameterization. Finally, the eight best‐fitting scenarios were repeated with the additional two breed sets as replicates.

TreeMix (Pickrell & Pritchard, 2012) was used to address any ambiguity between ABC models differing by only migration. The three replicate breed sets used in the ABC analyses were merged together, and a maximum‐likelihood tree was constructed using TreeMix under default parameters. Iteratively, weighted migration edges were added to the network, until 99.8% of the variance of the ancestry between the populations was explained by the model.

## 3 RESULTS

### 3.1 Admixture and population structure

**Figure 3:**
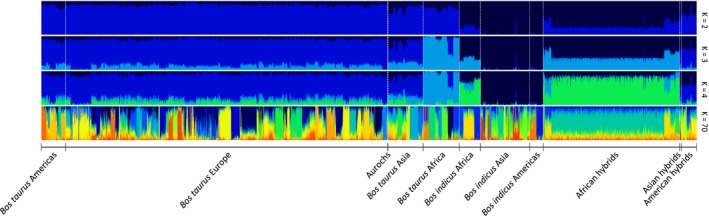
Admixture individual assignment plots for 179 cattle populations and one auroch sample for K = 2, 3, 4 and 70. Each vertical bar represents an individual, and the proportion of each colour in that bar corresponds to the ancestry (genetic variation) of an individual deriving from a given cluster

Admixture analysis of 180 populations was performed for 61 different values of K, ranging between 1 and 150. The CV error across all individuals was lowest at K = 70 (0.545) (Supporting Information Figure S2); however, 56% of the reduction in CV error (CV error difference 0.06) was observed from K 1 to 5. At K = 2, the main separation was between *B. taurus* and *B. indicus* (Figure 3, for more information on the breeds see Supporting Information Figure S3), while for K = 3 the African taurine breeds formed a separate cluster from the European taurine populations (Figure 3). K = 4 showed the split between Asian and African indicine breeds (Figure 3). At higher levels of K, many taurine breeds could be differentiated from each other, whereas for indicine breeds, the main differentiation was observed for Asiatic breeds, while the African indicine breeds were more similar to each other, presenting a similar level of admixture (data not shown).

**Figure 4:**
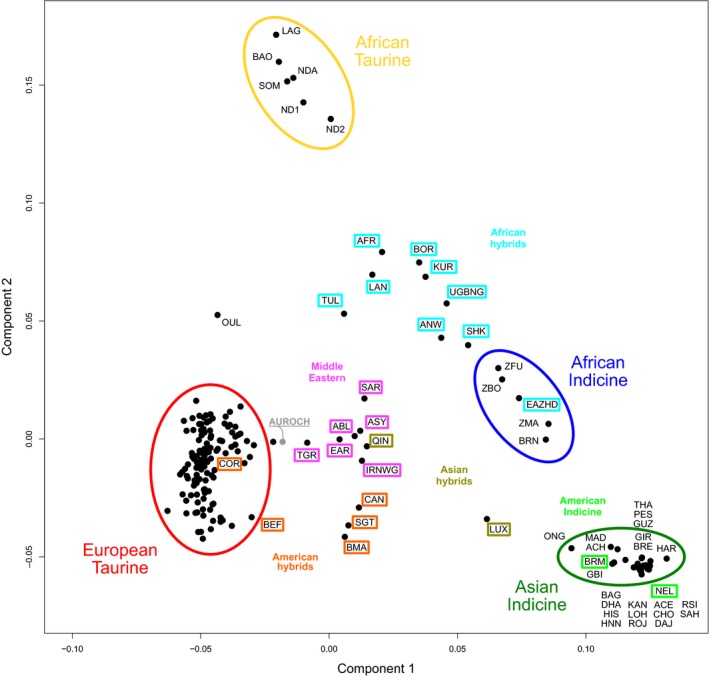
Multidimensional scaling (MDS) plot of 3,197 individuals belonging to 180 populations of *Bos primigenius primigenius*,* Bos indicus, Bos taurus* and hybrids

**Figure 5:**
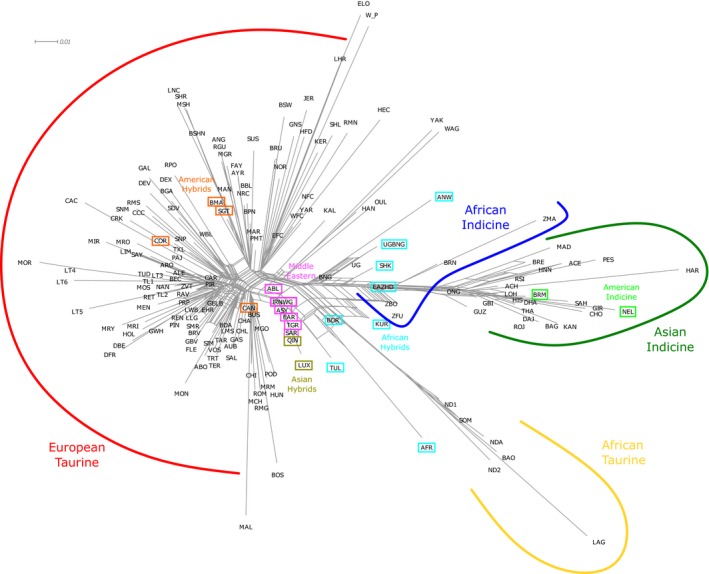
Neighbour‐net using *F*_ST_ distances for 174 populations of *Bos indicus, Bos taurus* and hybrids featuring more than one sample (see Supporting Information Table S1 for label information). Scale for *F*_ST_ distance is displayed in the top left

Multidimensional scaling analyses resulted in the two major axes explaining ~31% and ~15% of the variance, respectively. The first component separated taurine from indicine cattle, with hybrid breeds occurring in the middle between the two main cattle groups, while the second component separated African taurine from the rest of the cattle breeds (Figure 4). The African hybrids between taurine and indicine breeds were placed between the African taurine and the African indicine populations, while the Asian hybrids were placed between the European taurine and Asiatic indicine cattle. These results were supported by the breed clustering observed in the neighbour‐net analysis, which clearly separated Eurasian taurine, African taurine and the indicine breeds. African indicine cattle occurred between the Asiatic indicine populations and the area of the network where the taurine–indicine hybrids were clustered (Figure 5). Overall, both analyses depicted two main clusters of hybrids between *B. taurus* and *B. indicus* (Figures 4 and 5): (a) African hybrids (AFR, ANW, BOR, BRN, EAZHD, KUR, LAN, SHK, TUL, UGBNG), closer to African indicine breeds; and (b) American hybrids (BEF, BMA, CAN, COR, SGT), closer to European taurine cattle. More intermediate positions were assigned to Asian hybrids (LUX, QIN). Apart from hybrids, the Middle Eastern breeds (IRNWG, TGR, EAR, ABL, ASY, SAR) showed a slightly higher proximity to the Eurasian taurine component (Figures 4 and 5). Creole cattle (CCC, CRK, RMS, SNM, SNP, TXL) were placed in the European taurine group, while the American indicine breeds (BRM, NEL) clustered along Asian indicine populations (Figure 5). Finally, the aurochs was placed between the European and Middle Eastern taurine groups.

### 3.2 Approximate Bayesian computation (ABC)

Approximate Bayesian computation was used to reconstruct the demographic history of taurine and indicine breeds. We first assessed whether the reduced data set (2K) resulted in qualitatively the same admixture pattern as the larger data set (8K). The CV error values for K from 1 to 5 were similar for both data sets (less than 1% difference for K = 3 and K = 4; Supporting Information Figure S4). For K = 4, each breed was assigned to one cluster with the ZebuAF showing admixture partially deriving from TaurAF but mostly from ZebuAS (Supporting Information Figure S4), while for K = 3 the clusters corresponded to TaurAf, TaurEU and ZebuAS. Both results reflected the divergence patterns observed in the population structure analyses.

Of the 34 summary statistics originally chosen for the ABC analyses, 17 were removed because of correlations with other statistics, for example, mean number of alleles per locus and pairwise differences between populations due to the high correlation with heterozygosity (Supporting Information Table S3; Figure S5). The remaining 17 summary statistics included measurements of diversity (e.g., mean and standard deviation of heterozygosity across loci for each population) and pairwise divergence (e.g., mean and standard deviation of *F*_ST_; Supporting Information Table S3). Each observed summary statistics was within the 95% quantiles of the simulated data from that summary statistics, consistently across all scenarios and replicates.

**Figure 6:**
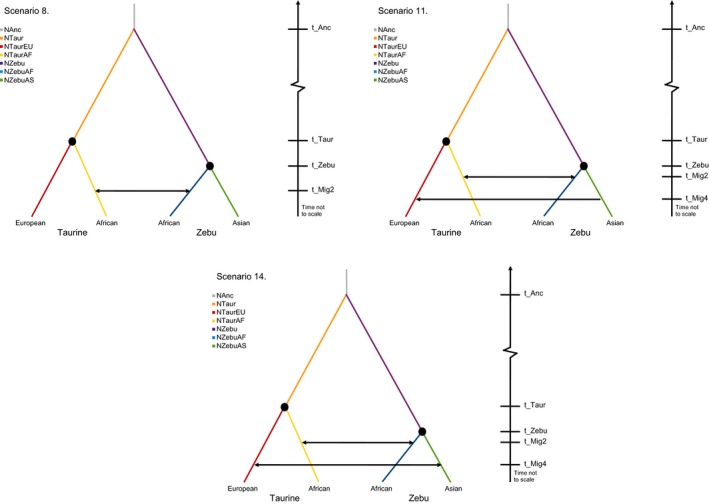
The three best‐modelled scenarios for the domestication history of cattle using approximate Bayesian computation (ABC). Domestication episodes and migratory events between populations are shown by black circles and arrows, respectively

**Figure 7:**
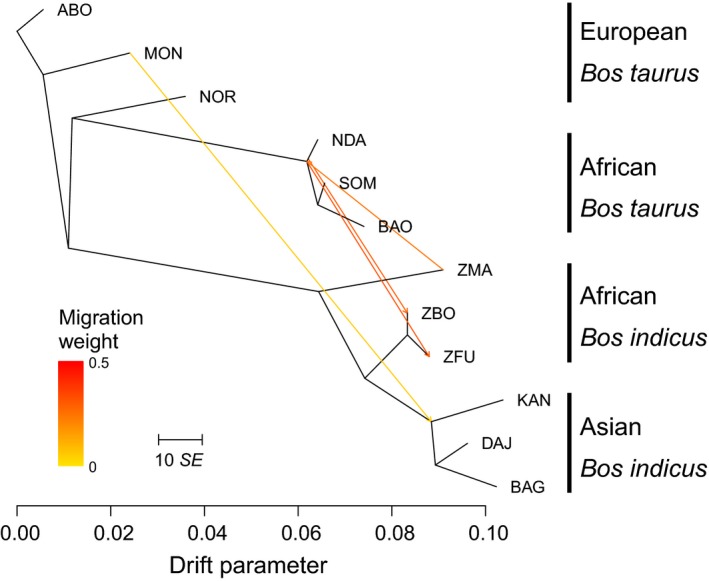
Phylogenetic network of the inferred relationships between 12 cattle breeds estimated using TreeMix. Migration edges between breeds are shown as arrows pointing towards the recipient population and coloured according to the proportional ancestry received from the donor population. Scale bar is 10 times the mean standard error of the estimated entries in the covariance matrix

The test of the first four scenarios showed a BF > 3 when comparing scenarios 1 and 2 with two domestication events, over scenario 4, which included three episodes of domestication (scenario 3 with two domestication events showed the lowest MD and *p*‐value, reflecting its unsuitability to represent the observed genetic data). Although additional models with three domestication events were tested, scenarios with only two domestications were better supported than those with three domestication events in general (Table 2). Of the 15 modelled scenarios, initial testing showed low support for scenarios 1 to 7 and therefore was not replicated. Among the replicated scenarios, three were identified consistently as best fitting across the three breed sets tested (in bold in Table 2). Scenario 8 displayed high MD and *p*‐values, with the following features: (a) domestication occurred on two independent occasions and (b) a bidirectional migration event between TaurAF and ZebuAF. The other two scenarios with highest MD and *p‐*values built on scenario 8 with the addition of a unidirectional (scenario 11) or bidirectional (scenario 14) migration between ZebuAS and TaurEU (Figure 6). The MD difference between these three scenarios was too small to discriminate between them (i.e., BF < 3; Table 2), with estimates of the posterior distributions of parameters such as the effective population size remaining relatively consistent across all three scenarios. These effective population size estimates suggest that following the divergence between the lineages that led the *B. taurus* and *B. indicus* (~250,000 YA) the ancestral taurine and indicine lineages grew demographically from ~10^3^ until reaching effective population sizes of ~10^5^ to 10^6^; however, between ~3,600 and 7,900 generations ago these reduced until reaching ~10^4^ (Supporting Information Table S4). The TreeMix analysis recovered the expected phylogenetic relationships between the combined breed sets before migration edges were added. Four migration edges were added before the model explained over 99.8% of the variance of the ancestry between the twelve populations (Figure 7). The first two edges were directed from TaurAF to ZebuAF, each weighted between 0.29 and 0.32 of the proportional ancestry received from the TaurAF into ZBO and ZFU. Additionally, a migration edge occurred in the reverse direction between ZebuAF (ZMA) and TaurAF at a similar strength (0.24). Finally, a weak (0.05) migration edge from TaurEU (MON) into the ancestor of ZebuAS was also observed.

**Table 2:** Approximate Bayesian computation (ABC) results for different hypothesized domestication histories within taurine (*Bos taurus*) and indicine (*Bos indicus*) cattle

Scenario	Breed set 1^a^		Breed set 2^a^		Breed set 3^a^		Domestication Events	Scenario Description^c^
	MD^b^	*p‐value* b	MD^b^	*p‐value* b	MD^b^	*p‐value* b		
1	13,685.50	0.29					2	*B. t*. domestication at the time of *B. t*. divergence, *B. i*. domestication at the time of *B. i*. divergence.
2	1,688.92	0.77					2	*B. t*. domestication at time of *B. t*. divergence, *B. i*. domestication before *B. i*. divergence.
3	22.14	0.15					2	Domestications each before divergence of *B. t*. and *B. i*.
4	325.70	0.73					3	*B. t*. domestications after *B. t*. divergence, *B. i*. domestication before *B. i*. divergence.
5	0.51	0.08					3	Scenario 4 with ancient *B. t*. divergence (lower bound at 12,500 generations)
6	1,059.53	0.97					3	*B. t*. domestications after *B. t*. divergence, *B. i*. domestication at the time of *B. i*. divergence.
7	5,747.75	0.26					2	Scenario 1 with bidirectional migration before either divergence.
**8**	**578,215.00**	**0.82**	**531,478.00**	**0.66**	**237,680.00**	**0.46**	**2**	**Scenario 1 with bidirectional migration between African ** ***B. t.*** **and African ** ***B. i.*** **after both divergences**.
9	329,323.00	0.77	991,43.70	0.58	100,88.40	0.61	2	Scenario 8 with bidirectional migrations before either divergence.
10	244,663.00	0.77	273,67.20	0.55	321,27.50	0.49	2	Scenario 8 with unidirectional migration from African *B. i*. to European *B. t*. after both divergences.
**11**	**360,715.00**	**0.86**	**592,151.00**	**0.75**	**491,523.00**	**0.70**	**2**	**Scenario 8 with unidirectional migration from Asian ** ***B. i.*** **to European ** ***B. t.*** **after both divergences.**
12	210,936.00	0.78	327,979.00	0.62	231,650.00	0.50	2	Scenario 8 with unidirectional migration from African *B. i*. and Asian *B. i*. to European *B. t*. after both divergences.
13	2.37	0.00	598.91	0.14	22.44	0.06	2	Scenario 8 with constant ongoing bidirectional migration between *B. t*. and *B. i*. before either divergence
**14**	**194,460.00**	**0.83**	**673,869.00**	**0.84**	**620,562.00**	**0.72**	**2**	**Scenario 8 with bidirectional migration between Asian ** ***B. i.*** **and European ** ***B. t.*** **after both divergences.**
15	124,482.00	0.66	235,974.00	0.68	364,806.00	0.58	2	Scenario 14 with ancient bottlenecks within *B. t*. and *B. i*.

Note

The three best scenarios with consistently high MD values across breed sets are highlighted in bold. Models are shown in Figures 2, 6 and Supporting Information Figure S1.

^a^Breed set 1: Normande (NOR), Somba (SOM), Zebu from Madagascar (ZMA), Kankraj (KAN); Breed set 2: Abondance (ABO), N'dama (NDA), Zebu Fulani (ZFU), Bhagnari (BAG); Breed set 3: Montbeliard (MON), Baoule (BAO), Zebu Bororo (ZBO), Dajal (DAJ). ^b^Marginal density (MD) and *p‐*values are taken from the 1,000 simulations most similar to the observed data from 1 million simulations. ^c^Divergences here refer to events within *Bos taurus* (*B. t*.) and *Bos indicus* (*B. i*.) rather than the ancient divergence between them from *Bos primigenius primigenius*.

## 4 DISCUSSION

Success in domesticating many species of animals and plants facilitated the gradual change in human societal behaviour, shifting from nomadic foragers and hunters to a sedentary farming society (Diamond, 2002). Cattle provide humans with food, hides and draught power for farming, transport and construction. Animal husbandry and food production may have contributed to the first uneven divisions of labour within early human societies (Ajmone‐Marsan et al., 2010). The associated population growth would have led to increased demographic pressure and encouraged dispersal of human populations out of the domestication centres, often accompanied by cattle (Taberlet, Coissac, Pansu, & Pompanon, 2011), helping farmers to colonize habitats that were unavailable to indigenous hunter‐gatherers. Our worldwide survey on 180 populations using SNP array data depicted genetic ancestries, admixture, introgression and migration patterns of cattle at a global scale, and gained insights into the long‐standing debate on the existence of a third domestication event from aurochs in north‐east Africa. However, there are potential issues regarding ascertainment bias associated with the SNP arrays used in this study (Gautier et al., 2010; Matukumalli et al., 2009; Orozco‐terWengel et al., 2015), as reflected by the significantly higher genetic variation within taurine cattle when compared to indicine breeds. Higher *H*_o_ in European taurine populations and a potential overestimation of the inbreeding coefficient (*F*_IS_) in indicine cattle would be an unexpected result had the data set used an array not biased towards *B. taurus* variation (Supporting Information Table S1). Ascertainment bias was reduced through removing a proportion of the markers in LD, and highly correlated markers with similar genealogical history were removed from the data set, reducing multicollinearity effects that would lead to overestimation of differentiation measurements. LD pruning of SNPs has been shown to be effective at reducing heterozygosity overestimations when compared to whole‐genome sequencing and, although this methodology underestimates *F*_ST_, it is consistent regardless of relatedness to the ascertainment populations (Malomane et al., 2018). Furthermore, by replicating the ABC scenarios to increase the sampling scheme, we attempted to reduce any breed specific biases and more effective at capturing the demographic history representative of whole subgroups (Rougemont et al., 2016).

The Admixture analysis best supported the partition for K = 70; however, the low variation in CV error for K values between 15 and 100 suggests that sample groupings above K = 15 only reflect minor improvements in the resolution of the clusters detected. This observation is likely to be an outcome of several factors influencing the statistical power to discriminate between the populations analysed, for example, the presence of known hybrids in the data set, low sample sizes for some breeds (*n *<* *8), or the presence of related individuals (e.g., family structure) (Barbato et al., 2017; Kijas et al., 2012; McTavish et al., 2013; Orozco‐terWengel et al., 2015). This effect may be further exacerbated due to shared genetic variation and incomplete lineage sorting among populations, reflecting their low divergence since they were founded ~200 YA (Bradley et al., 1996). An additional factor influencing the power to separate populations is the presence of gene flow between them, as reflected by the admixture signals in African indicine cattle. Deriving from Asiatic indicine populations introduced to Africa ~2,500–3,500 YA, African indicine cattle represent an important component of African taurine genetic diversity, probably reflecting gene flow between these populations due to their geographic proximity, as well as deliberate hybridization by farmers to incorporate local adaptations (e.g., trypanotolerance) into the indicine animals following their entry into Africa (Troy et al., 2001). Analyses with Admixture for low K values initially divided taurine and indicine cattle (K = 2; Figure 3), as expected due to their relatively ancient divergence from their common ancestor, *B. p. primigenius*. Increasing K to 3 and 4 caused the within‐species division of taurine into African and Eurasian and of indicine into African and Asian genomic components, respectively (Figure 3). Interestingly, for low clusters (K = 2–4), African hybrids display similar proportional ancestry to African indicine. The introgression of indicine into African taurine cattle is predicted to have initially occurred ~4,000 YA, resulting in a consistently high indicine component within African hybrid cattle (Bahbahani et al., 2017), possibly reinforced by modern admixture with African indicine.

The neighbour‐net and MDS analyses identified similar patterns of divergence between indicine and taurine populations (Figures 4 and 5). The first two components of variation in the MDS analysis also identified the taurine vs. indicine (~31%) and African vs. Eurasian taurine (~15%) splits. While the division between the taurine groups explained much less genetic variance than the first component, it still explained a substantial proportion of the total (and more than twofold the third component), suggesting substantial differentiation between the two taurine groups, consistent with the hypothesis that the two groups harbour rather distinct genetic pools. In contrast to this, African indicine populations strongly resembled Asiatic indicine breeds. The relatively short distances between African indicine to both Asian indicine and African taurine populations in the neighbour‐net suggest admixture between these groups. Furthermore, the central block of the neighbour‐net depicts multiple complex connections among breeds, suggesting relatively recent divergence across many populations (Felius, 1995). Despite being classified as *B. taurus*, Middle Eastern breeds occupy central positions both in the neighbour‐net and MDS analyses, suggesting an admixed genetic background with an indicus component, as has been observed for several breeds from the Middle East and Fertile Crescent (Decker et al., 2014; Karimi et al., 2016; Loftus et al., 1999). Consistent with the hypothesis of admixture of European taurine populations with local aurochs prior to their extinction approximately 400 YA (Achilli et al., 2008; Park et al., 2015; Upadhyay et al., 2017), the *B. p. primigenius* sample in this study is placed close to Eurasian taurine groups in the MDS analysis. Although Decker et al. (2014) suggested a significant admixed aurochs ancestry for African taurine populations, with aurochs contributing up to 26% of their genomes, our results failed to detect this influence, almost certainly because the British aurochs used is not an appropriate proxy for African aurochs—often classified as *B. p. africanus* or *B. p. opisthonomous* (Clutton‐Brock, 1999). Similar to the Admixture results (Figure 3), African hybrids from *B. taurus* and *B. indicus,* that were expected to be found in the central areas of the neighbour‐net and MDS graphics, were placed near to the African indicine group, pointing towards a higher contribution of the African indicine genepool in the formation of those hybrid breeds, as opposed to American hybrids that displayed higher influences from European taurine breeds. The genetic composition of Asian hybrids (QIN, LUX) was more balanced between Asian indicine and taurine origins. As expected, Creole cattle were placed closer to the European taurine cluster (Decker et al., 2014; Martínez et al., 2012; Pitt et al., 2018), and American indicine cattle clustered with Asian indicine breeds (Orozco‐terWengel et al., 2015).

The substantial differentiation between the European and African taurine populations when compared to African and Asian indicine groups may be explained by several factors, such as lower gene flow between Africa and Europe than between Africa and Asia or a more recent divergence of indicine cattle. Another explanation may be a stronger effect of genetic drift in African taurine populations with respect to Eurasian taurine or indicine populations. Genetic drift would increase the rate of the change in allele frequencies in populations with a smaller effective population size (*N*_e_), increasing the probability of accumulating differences between populations. An analysis of the trajectories of the effective population size over the last ~8,000 years shows that most cattle populations around the world exhibit relatively large effective population sizes at the beginning of the Holocene (~3,000 *N*_e_); however, more recently, all cattle populations have experienced a drastic decrease in *N*_e_ reaching modern *N*_*e*_ values of ~500 or less for many populations (Barbato, Orozco‐Terwengel, Tapio, & Bruford, 2015; Orozco‐terWengel et al., 2015), with the African taurine population showing, on average, larger *N*_e_ values than Eurasian taurine populations, albeit only marginally.

The hypothesis of an additional domestication event in Africa from local aurochs has been also proposed to explain the large difference between taurine branches. We explicitly tested the hypothesis of whether three domestication events better explain the genetic variation observed in modern domestic cattle when compared to the more widely accepted two domestication scenario. ABC tested on a number of demographic scenarios showed reasonable support for three domestication events (scenarios 4 and 6) in terms of how well the estimated GLM fitted the observed data (i.e., the *p‐*value). However, when scenarios including three domestications were compared to similar models in which only two domestications occurred, the latter received stronger support (BF > 3) (Table 2). The rejection of a hypothesized third domestication suggests an alternative explanation for the differences observed between African and Eurasian taurine populations: admixture between migrating domestic cattle from the Middle East and indigenous African. Domestic cattle and their wild relatives occupied the same geographic regions for a long period of time, which raises the possibility that both taurine and indicine cattle naturally hybridized with aurochs or that local farmers mixed them with local aurochs to restock their herds (McTavish et al., 2013; Troy et al., 2001). Uniparental loci such as mtDNA and Y‐chromosome studies have generally underplayed the significance of admixture with wild aurochs, as haplotypes present in ancient DNA samples are often closely related but phylogenetically distinct from those in extant cattle samples (Edwards et al., 2010; Götherström et al., 2005; Schibler, Elsner, & Schlumbaum, 2014; Troy et al., 2001). However, there is evidence of gene flow from wild aurochs prior to the extinction (~400 YA) into extant cattle in areas such as Italy, Iberia, southern Europe and the British Isles (Achilli et al., 2008; Park et al., 2015; Upadhyay et al., 2017). These results are consistent with those in other domesticated animals such as horses (Warmuth et al., 2012), pigs (Frantz et al., 2015) and camels (Almathen et al., 2016), where gene flow with wild populations has been observed in modern breeds.

Previous studies using SNP data genotyped in taurine and indicine cattle have found an African taurine component in European taurine cattle reaching an average of 10% of their genotype, while between 5% and 10% of the genetic background in some European breeds is of indicine origin (e.g., Decker et al., 2014; McTavish et al., 2013). We used ABC to test alternative migration hypotheses between the sets of African and Eurasian taurine and indicine breeds used to model the domestication events. However, it was not possible to differentiate between a scenario with bidirectional gene flow between African breeds, and two alternative ones that further included unidirectional or bidirectional gene flow between the Eurasian breeds. Further specification of the model was unsuccessful and trying to define the presence or manner of migration between other groups only yielded insignificant changes between models. This could be due to the difficulty in accurately reconstructing demographic history and model differentiation, for example, divergence and gene flow can be indistinguishable to isolation followed by secondary contact, or brief bottlenecks remaining unidentified if preceded by large expansions due to the increased genetic diversity (Gray et al., 2014; Rougemont et al., 2016). These limitations restrict the intrinsic complexity in the models, ideally, events such as divergences and domestication would be reconstructed as gradual processes occurring over many generations as cattle were shifted from prey animals, to managed herds, to captive bred stock (Larson & Burger, 2013). Despite efforts to limit overparameterization by reducing the ranges of many parameters, both the number of prior parameters and their range may have reduced statistical power of posterior estimates (Wegmann et al., 2010). The progression of refining the ABC model through serial selection and diversification of preferentially chosen scenarios was the most computationally efficient methodology to define increasingly more complex models. “Less favourable” models were rejected at each step to constrain the otherwise very large number of models simulated. Although this saves significant time and resources, it is built on the assumption that discarded models will never be viable regardless of alterations; unfortunately, this compromise, along with the reduction in the data set from 8K to 2K, was necessary logistical restrictions. However, the first three migration edges incorporated into the maximum‐likelihood tree generated in TreeMix recaptured the same migratory patterns between TaurAF and ZebuAF as tested in ABC scenario 8 (Figures 6 and 7) and explained up to 99.78% of the variance in the tree. Adding a fourth migration edge marginally increased the explanation of the variance in the tree and corresponded to a weak migration edge from TaurEU into ZebuAS. Overall, this supports the ABC results and indicates that the more important gene flow between populations occurred between *B. taurus* and *B. indicus* within Africa, although minor movements of cattle between Europe and Asia may also contribute to shape the population structure we see today.

In conclusion, SNP array data collected in more than three thousand cattle samples belonging to 180 populations with a worldwide geographic distribution encompassing Africa, Europe, Asia and the Americas continents provided a comprehensive picture of genetic diversity, population structure and demographic dynamics of cattle populations at a global level. The analyses confirm the large differentiation between African and Eurasian taurine and the high levels of admixture in African indicine cattle from both Asian indicine and African taurine cattle, and reveal a higher contribution from African indicine genetic origin in the formation of African hybrids, as opposed to American hybrids which exhibited a higher influence from taurine breeds. Modelling the domestication history of cattle using approximate Bayesian computation consistently favoured scenarios involving only two domestication events, discarding a third *B. p. primigenius* domestication in Egypt and suggesting the subsequent hybridization from local aurochs to explain the additional genetic variation detected. Further analysis exploring more domestication situations, particularly focussed on migration between groups (e.g., African indicine and European taurine cattle), will help to disentangle the complex human‐mediated microevolution of domestic cattle, in particular if using data deriving from whole‐genome sequence data that suffer less of ascertainment bias as SNP arrays do. Paleogenomics analyses of Middle Eastern and African wild aurochs predating domestication and early Middle Eastern and African domestic cattle will provide the data required to fully address these questions.

## CONFLICT OF INTEREST

None declared.

## Supporting information


** **

** **

** **

** **

** **

** **

** **

** **

** **
