# Supplementary figures and images for: Domestication of cattle: Two or three events?
Source: Evol Appl. 2018 Jul 23;12(1):123–36. doi: 10.1111/eva.12674 (PMC6304694; doi:10.1111/eva.12674)

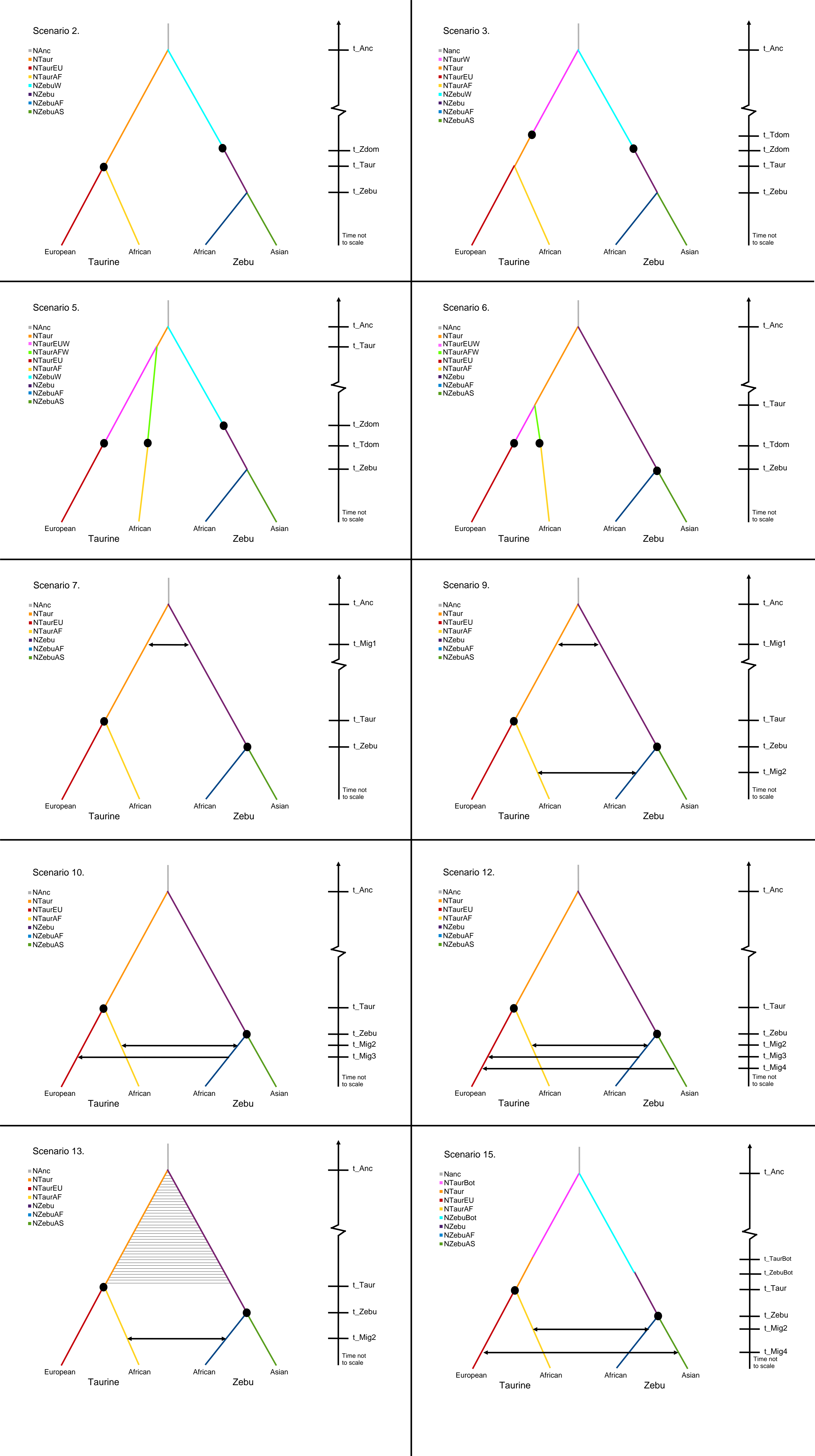

Supplement: Supplementary file 1 [file EVA-12-123-s001.pdf]

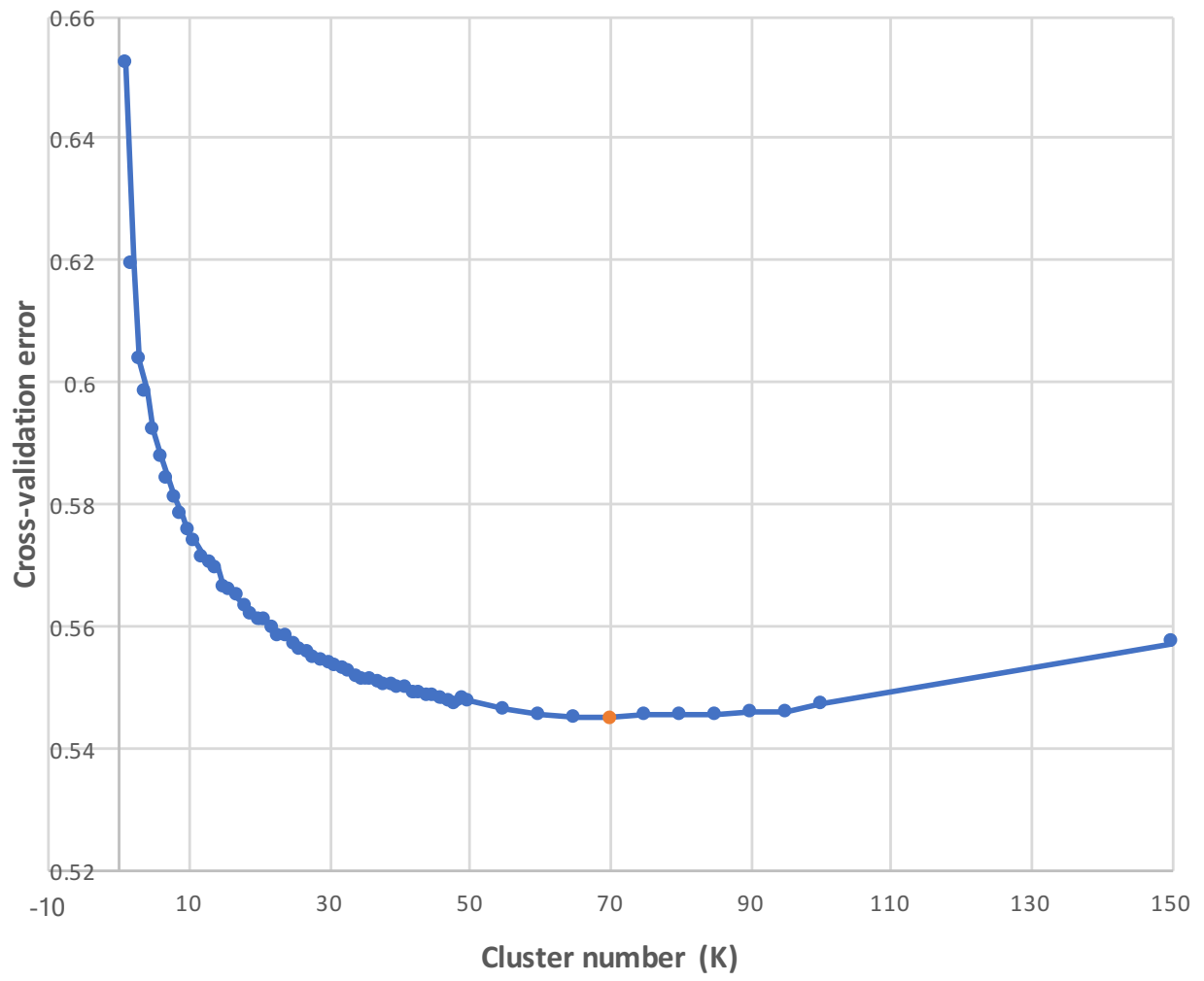

Supplement: Supplementary file 2 [file EVA-12-123-s002.pdf]

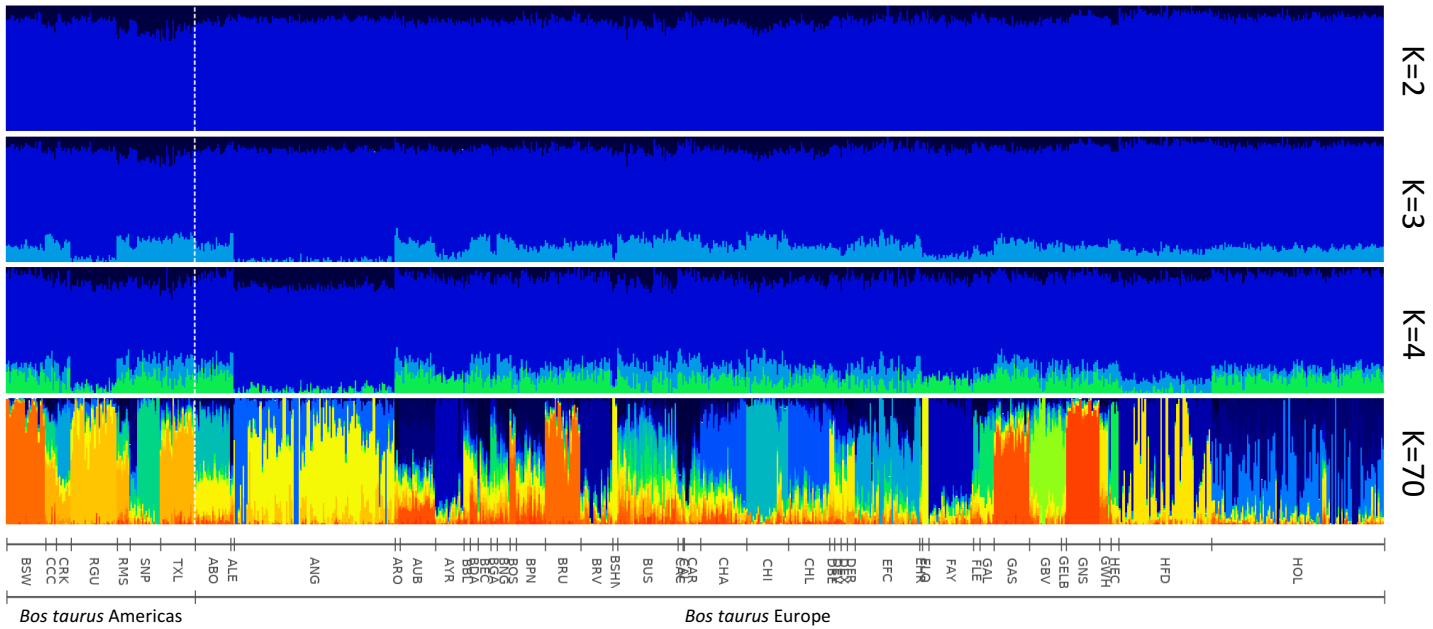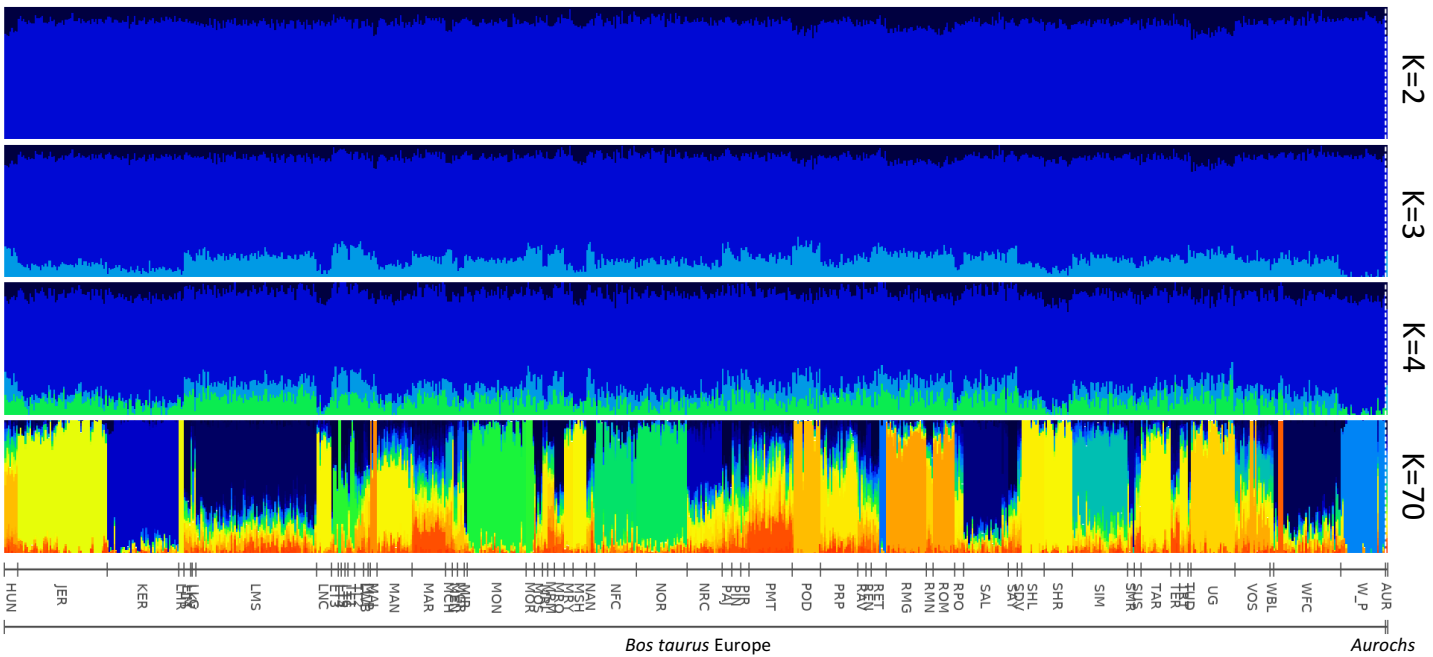

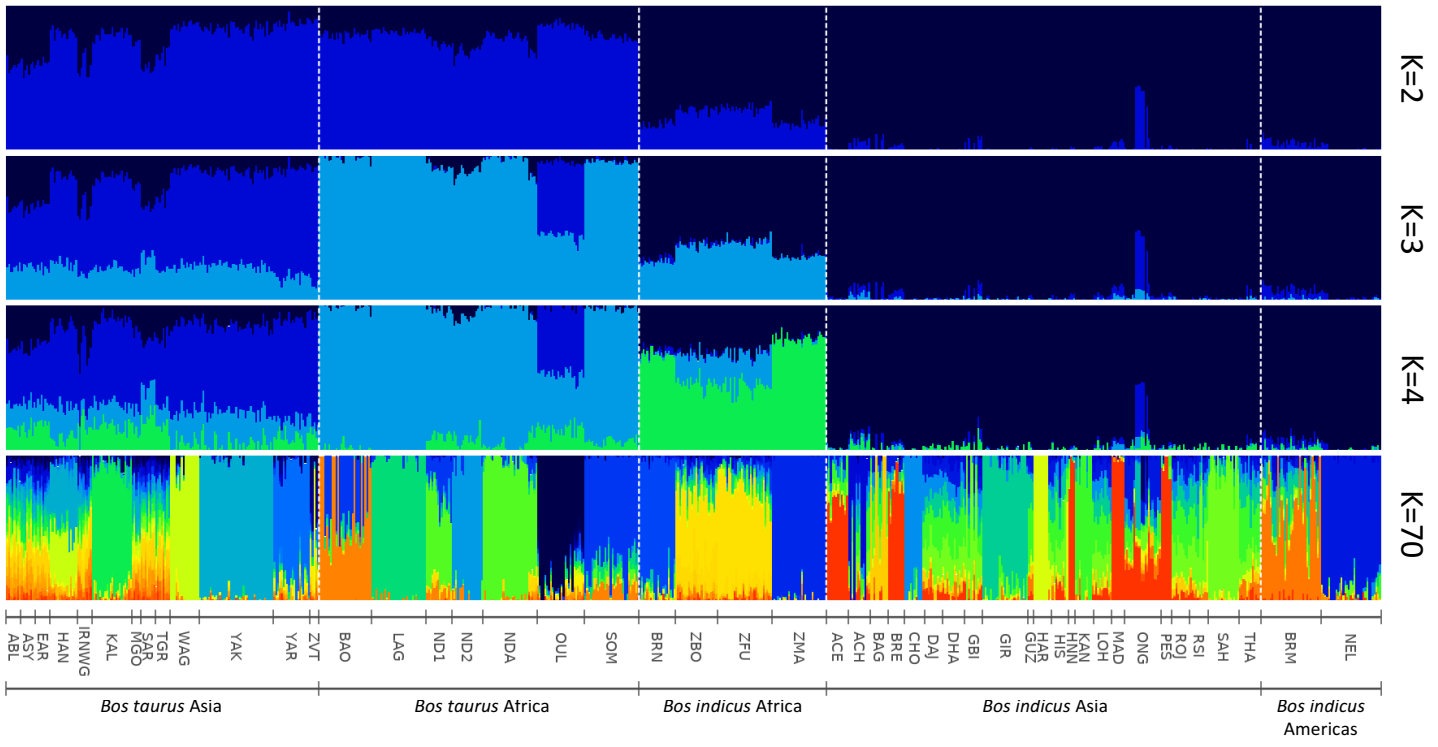

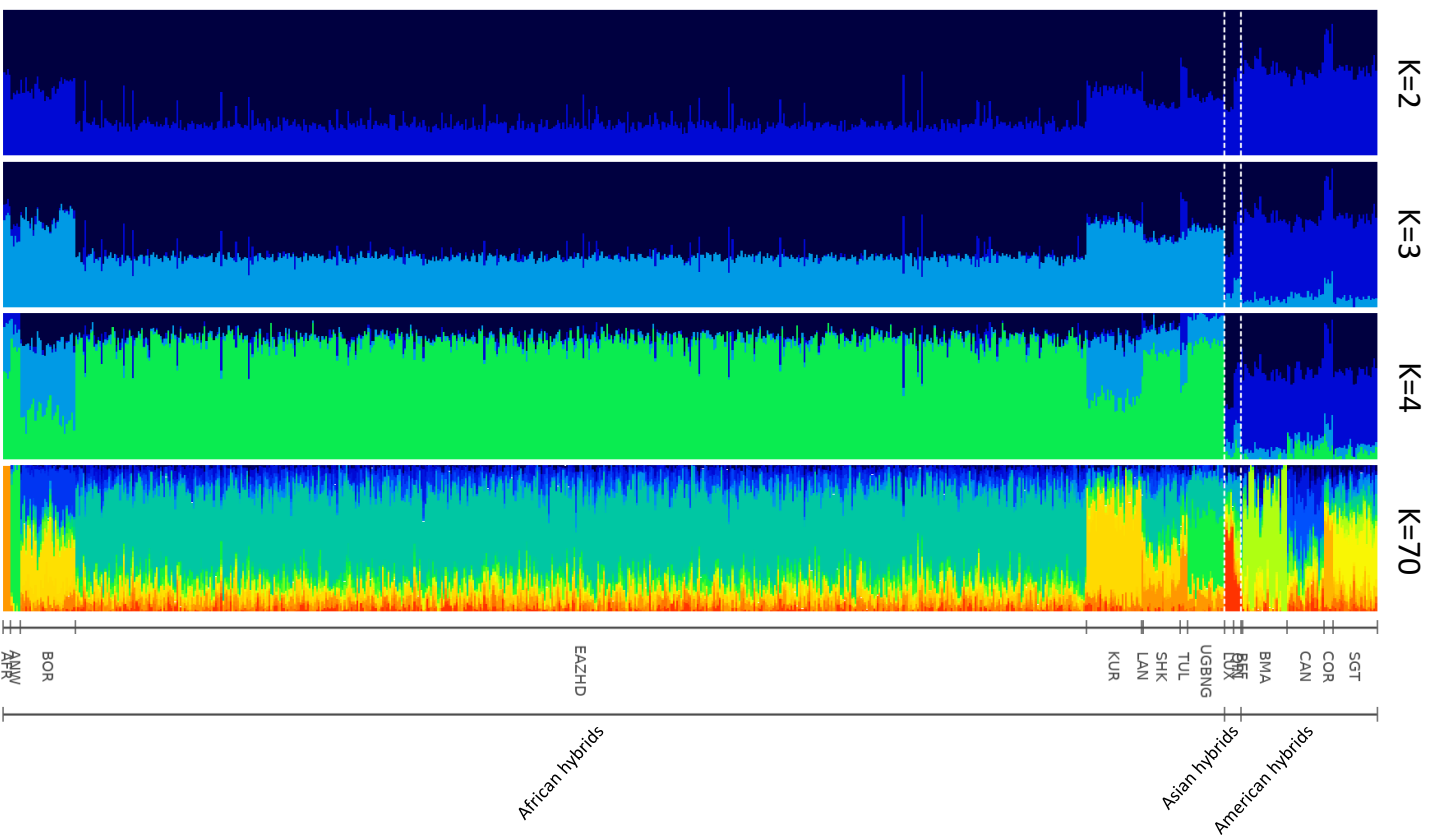

Supplement: Supplementary file 3 [file EVA-12-123-s003.pdf]

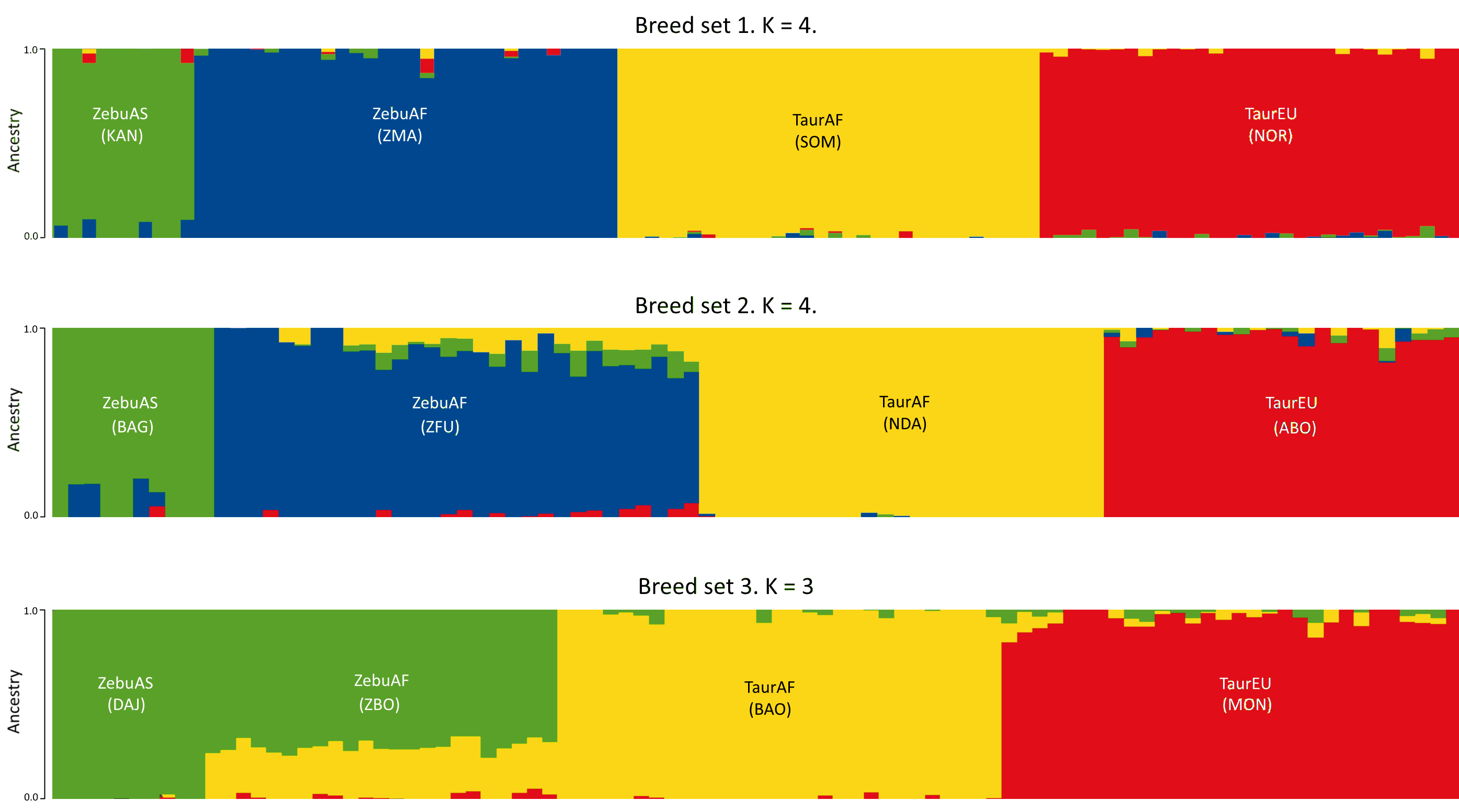

Supplement: Supplementary file 4 [file EVA-12-123-s004.png]

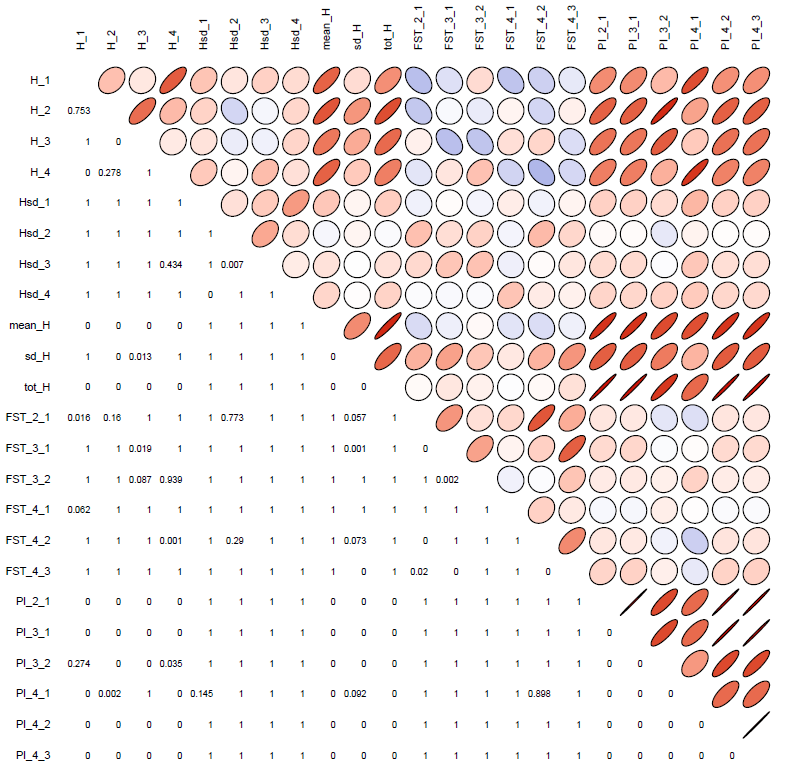

Supplement: Supplementary file 5 [file EVA-12-123-s005.png]
